# Lipid Peroxide-Derived Reactive Carbonyl Species as Mediators of Oxidative Stress and Signaling

**DOI:** 10.3389/fpls.2021.720867

**Published:** 2021-10-28

**Authors:** Md. Sanaullah Biswas, Jun’ichi Mano

**Affiliations:** ^1^Department of Horticulture, Bangabandhu Sheikh Mujibur Rahman Agricultural University, Gazipur, Bangladesh; ^2^Science Research Center, Yamaguchi University, Yamaguchi, Japan

**Keywords:** environmental stress responses, plant hormone signaling, reactive electrophile species, reactive oxygen species, redox signaling

## Abstract

Oxidation of membrane lipids by reactive oxygen species (ROS) or O_2_/lipoxygenase leads to the formation of various bioactive compounds collectively called oxylipins. Reactive carbonyl species (RCS) are a group of oxylipins that have the α,β-unsaturated carbonyl structure, including acrolein and 4-hydroxy-(*E*)-2-nonenal. RCS provides a missing link between ROS stimuli and cellular responses in plants via their electrophilic modification of proteins. The physiological significance of RCS in plants has been established based on the observations that the RCS-scavenging enzymes that are overexpressed in plants or the RCS-scavenging chemicals added to plants suppress the plants’ responses to ROS, i.e., photoinhibition, aluminum-induced root damage, programmed cell death (PCD), senescence, abscisic acid-induced stomata closure, and auxin-induced lateral root formation. The functions of RCS are thus a key to ROS- and redox-signaling in plants. The chemical species involved in distinct RCS signaling/damaging phenomena were recently revealed, based on comprehensive carbonyl determinations. This review presents an overview of the current status of research regarding RCS signaling functions in plants and discusses present challenges for gaining a more complete understanding of the signaling mechanisms.

## Introduction

**FIGURE 1:**
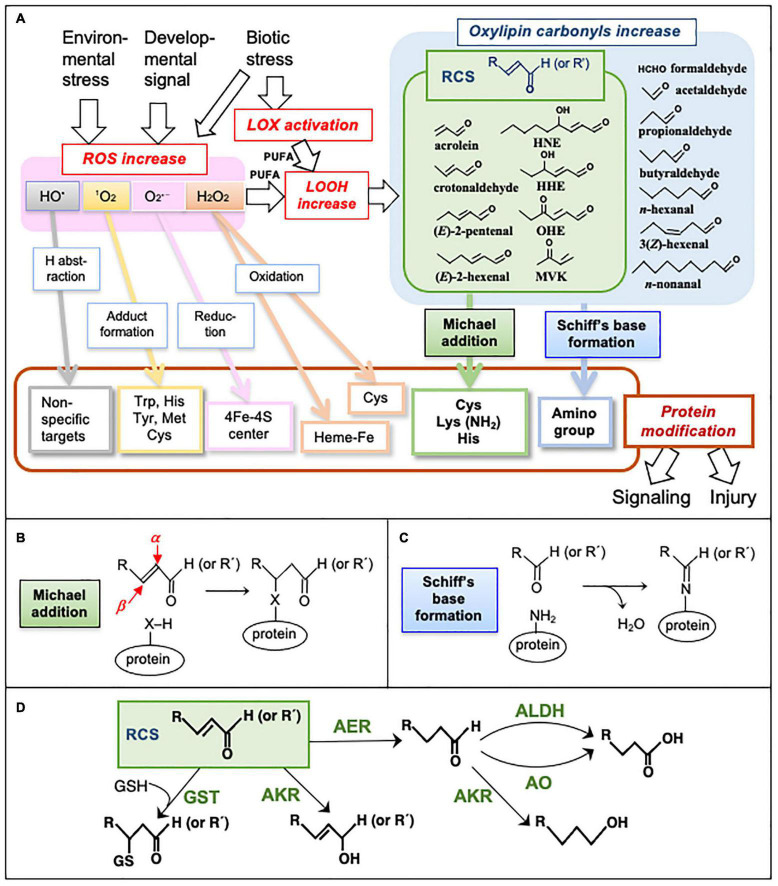
Metabolism and reactions of oxylipin carbonyls. **(A)** Formation of ROS and oxylipin carbonyls and their actions causing signaling and injury via protein modification. HO^•^ is highly reactive and non-specifically oxidizes almost all biomolecules. ^1^O_2_ is also highly reactive, and it prefers adduct formation on a double bond and a sulfur atom. O_2_^•–^, a relatively less reactive ROS, can reductively destroy the 4Fe-4S center in some enzymes such as aconitase (Halliwell and Gutteridge, 2015). H_2_O_2_ is also less reactive and may oxidize the Fe atom in the heme proteins such as ascorbate peroxidase or guaiacol peroxidase to inactivate them (Miyake and Asada, 1996). Oxylipin carbonyls and RCS, produced via the oxidation of PUFA by ROS, react with proteins in different manners from ROS. **(B)** Formation of the Michael adduct on a protein. The α- and β-carbons in an RCS molecule are indicated by red arrows. X, a nucleophilic atom. **(C)** Schiff’s base formation on a protein. **(D)** Enzymes to scavenge carbonyls and RCS in plants. AER, 2-alkenal reductase; AKR, aldo-keto reductase; ALDH, aldehyde dehydrogenase; AO, aldehyde oxidase; GST, glutathione transferase.

The production of reactive oxygen species (ROS) such as superoxide radical (O_2_^•–^), hydrogen peroxide (H_2_O_2_), and singlet oxygen (^1^O_2_) is intrinsically associated with redox reactions in aerobic cells (Halliwell and Gutteridge, 2015). In plants, intracellular ROS levels are changed by environmental stress, biotic stress, phytohormones, and developmental signals, and increases in ROS cause reprogramming of cells to enable them to survive in new conditions (oxidative signaling) or cell death (oxidative stress). Specifically, ROS are critical agents in determining cell fates in various physiological situations (Mittler, 2017; Mhamdi and Van Breusegem, 2018; Waszczak et al., 2018). Because ROS production sites are often associated with membranes (Asada, 2006), fatty acids [especially polyunsaturated fatty acids (PUFAs)] are oxidized by ROS to lipid peroxides (LOOHs). LOOHs are also produced via the lipoxygenase (LOX)-catalyzed oxygenation of PUFAs. Subsequently, LOOHs are metabolized or non-enzymatically decompose to a variety of aldehydes and ketones (collectively designated as “oxylipin carbonyls”) (Blée, 1998). A carbonyl group can form a Schiff’s base with an amino group and hence are potential protein modifiers (Figure 1).

Among the oxylipin carbonyls, those that have the α,β-unsaturated bond are grouped as reactive carbonyl species (RCS) (Mano, 2012; Mano et al., 2019a) or oxylipin reactive electrophile species (RES) (Farmer and Mueller, 2013) because they can form a Michael adduct with a nucleophilic group on proteins (Cys, His, or Lys residue) or nucleic acids (guanine base) due to the high electrophilicity of their β-carbon (Esterbauer et al., 1991). This chemical property gives RCS a potential ability to mediate ROS-initiated signals/damage to proteins, implying that RCS can cause both “eustress” and “distress” to organisms depending on their intracellular levels, as can ROS (Kranner et al., 2010). For animals, the physiological importance of RCS in oxidative stress was established in the 1990s (Esterbauer et al., 1991). The role of RCS was initially regarded as cell-damaging agents, and more recently their functions as cellular signals to regulate gene expression have been recognized (Schopfer et al., 2011; Bachi et al., 2013; Parvez et al., 2018).

For plants, the occurrence of a wide variety of carbonyls including RCS (Schauenstein et al., 1977) and the toxicity of carbonyl compounds are known (Reynolds, 1977), but their physiological functions have been investigated only sparsely until the 1990s (Gardner et al., 1990). The possibility that oxylipin carbonyls evoke physiological responses in plants was tested with experiments in which certain carbonyls were poured or fumigated on plants. Bate and Rothstein (1998) showed the (*E*)-2-hexenal, a C6 RCS, induced defense gene in *Arabidopsis thaliana.* Edward E. Farmar’s group demonstrated that acrolein and methylvinyl ketone (MVK), which are also types of RCS, induced distinct groups of genes (Alméras et al., 2003; Weber et al., 2004). The endogenous formation of RCS such as 4-hydroxy-(*E*)-2-nonenal (HNE) was detected in *Daucus carota* cultured cells and the bell pepper fruit (Deighton et al., 1997, 1999).

In those studies, oxylipin carbonyls were investigated as end products of the LOX-dependent formation of LOOHs in the context of plant responses to biotic stressors (e.g., infection and wounding) that activate LOXs. In contrast, in abiotic (environmental) stress studies, thiobarbituric acid-reactive substances (TBARS), which mainly detect the RCS malondialdehyde (MDA), were frequently employed as a marker of lipid oxidation due to ROS. The physiological effects of MDA and a variety of other RCS — including the more toxic acrolein and HNE — have not received broad attention until recently.

There has been substantial progress in our understanding of the functions of RCS in the plant stress responses during the past decade (Farmer and Mueller, 2013; Mano et al., 2019a). This progress is due to advances in the comprehensive analysis of carbonyls in plant tissues and the use of RCS-scavenging enzymes in transgenic plants (described below). The roles of RCS, ranging from signaling agents to cell-damaging toxins in a continuum of effects, have been discovered in a variety of physiological responses of plants (Table 1).

**TABLE 1:** Plant responses to oxidative stimuli that are relevant to RCS.

**Stimulus/response^a^**	**Plant species**	**Modulation of RCS**			**References**
		**Method^b^**	**RCS level changes*^c^***	**Physiological outcome**	
Drought stress/leaf damage	*Nicotiana tabacum*	OE of AKR	Suppression of increase in MDA (as TBARS)	Suppression of damage	Oberschall et al., 2000
Cd stress, chilling stress/leaf damage	*N. tabacum*	OE of AKR	Suppression of increase in MDA (as TBARS)	Suppression of damage	Hegedüs et al., 2004
Heat stress, MV/leaf damage	*N. tabacum*	OE of AKR	Suppression of increase in MDA (as TBARS)	Suppression of damage	Turóczy et al., 2011
Heavy metal stress (Cu, Cd), H_2_O_2_ stress/damage	*Arabidopsis thaliana*	OE of ALDH	Suppression of increase in MDA (as TBARS)	Suppression of damage	Sunkar et al., 2003
NaCl stress/damage	*A. thaliana*	KO of *ALDH3*	Enhanced increase in MDA (as TBARS)	Enhancement of damage	Kotchoni et al., 2006
Strong light/leaf damage	*N. tabacum*	OE of AER	Suppression of increase in acrolein and (*E*)-2-hexenal	Suppression of photoinhibition	Mano et al., 2010
AlCl_3_stress/root damage	*N. tabacum*	OE of AER	Suppression of increase in acrolein, (*E*)-2-hexenal, HNE and HHE	Suppression of damage	Yin et al., 2010
MV/leaf damage	*A. thaliana*	KO of *AOR*	Enhanced increase in acrolein	Enhancement of damage	Yamauchi et al., 2012
Heat stress/leaf damage	*Cyclamen persicum*	KD of *CpFAD7*	Suppression of increases in acrolein, (*E*)-2-hexenal and MVK	Suppression of leaf injury	Kai et al., 2012
H_2_O_2_/PCD of cultured cells	*N. tabacum*	carnosine added	Suppression of increases in acrolein and HNE	Suppression of PCD initiation	Biswas and Mano, 2015
Darkness/senescence of siliques	*A. thaliana*	KO of *AAO4*	Increases in acrolein and MDA	Senescence facilitated	Srivastava et al., 2017
ABA/stomata closure	*N. tabacum*	OE of AER	Suppression of increases in acrolein, (*E*)-2-hexenal, HNE and HHE	Inhibition of stomata closure	Islam et al., 2016
MeJA/stomata closure	*N. tabacum*	OE of AER	Suppression of increases in acrolein, HNE, HHE and (*E*)-2-heptenal	Inhibition of stomata closure	Islam et al., 2020
Auxin/LR formation	*A. thaliana*	carnosine added	Suppression of increases in acrolein, crotonaldehyde and HNE	Inhibition of LR formation	Biswas et al., 2019

*^a^ ABA, abscisic acid; LR, lateral root; MeJA, methyl jasmonate; MV, methylviologen; PCD, programmed cell death.*

*^b^ AAO4, Arabidopsis aldehyde oxidase 4; AER, 2-alkenal reductase; AKR, aldo-keto reductase; ALDH, aldehyde dehydrogenase; AOR, alkenal/one oxidoreductase; CpFAD, chloroplastic fatty acid desaturate; KD, knock-down; KO, knock-out; OE, overexpression.*

*^c^ HHE, 4-hydroxy-(*E*)-2-hexenal; HNE, 4-hydroxy-(*E*)-2-hexenal; MDA, malondialdehyde; MVK, methylvinyl ketone, TBARS, thiobarbituric acid-reactive substances.*

In this review, we present an overview of current research on RCS metabolism in plants (formation and scavenging) (section “Occurrence and Metabolism of Reactive Carbonyl Species in Plants”), the potential effects of RCS on plants (section “Response of Plants to Exogenously Added Reactive Carbonyl Species”) and plant functions (section “Physiological Roles of Endogenously Generated Reactive Carbonyl Species That Are Verified by the Measurement of Reactive Carbonyl Species Contents”), and the target proteins of RCS (section “Target Proteins of Reactive Carbonyl Species”). We then discuss present challenges in the elucidation of the RCS signaling mechanisms (and therewith the ROS- and redox signaling mechanisms) in plants.^1^

## Occurrence and Metabolism of Reactive Carbonyl Species in Plants

### Determination of Carbonyls in Plants

More than a dozen oxylipin carbonyls are present in plants (Schauenstein et al., 1977). Typical oxylpin carbonyls and RCS are shown in Figure 1. Although they are common as carbonyls, the chemical reactivities of distinct species are very different (LoPachin and Ganvin, 2014), as are their biological effects (Reynolds, 1977; Alméras et al., 2003; Weber et al., 2004; Mano et al., 2009; Yamauchi et al., 2015). For investigations of the roles of RCS, it is necessary to know their levels in tissues and their changes in response to various physiological stimuli. The measurement of TBARS (Steward and Bewley, 1980; Hodges et al., 1999) is generally thought to represent the MDA level, but this method is not specific to MDA, and it cannot detect many RCS that are more highly reactive, and thus biologically more active, than MDA (Esterbauer et al., 1991).

For a comprehensive carbonyl determination, a separation of the hydrazone derivatives of carbonyls by high-performance liquid chromatography (HPLC) is suitable (Esterbauer et al., 1989). Because carbonyls are unstable (they are prone to reduction by reductants contained in plant extract, oxidation by ROS and O_2_, and adduct formation with nucleophilic molecules), the extracted carbonyls should be stabilized by derivatization with 2,4-dinitrophenylhydrazine (DNPH). The resulting hydrazone derivatives of carbonyls can be sensitively determined spectrophotometrically (with an absorbance peak ranging from 300 to 380 nm) after separation on a reverse-phase HPLC column (Esterbauer et al., 1989). With a protocol optimized for the extraction of hydrophilic RCS from plant tissues, it is possible to determine the content of 19 types of carbonyls including the most toxic RCS acrolein and HNE (Mano and Biswas, 2018). More than a dozen types of oxylipin carbonyls were identified in leaves and roots, and their significant increases in response to stress treatments were observed (Mano et al., 2010; Yin et al., 2010). DNPH-derivatized carbonyls are also determined efficiently by liquid chromatography-mass spectrometry (LC-MS). The mass spectrometer is operated in negative ion mode with multiple reactions monitoring (MRM) (Roach et al., 2017). Significant increases in the levels of RCS such as acrolein, MDA, (*E*)-2-hexenal, and (*E*)-2-pentenal were detected in photoautotrophic *Chlamydomonas reinhardtii* LHCSR3-deficient strain *npq4* under high light conditions (500 μmol photons m^–2^ s^–1^) (Roach et al., 2017), on ^1^O_2_ exposure (Roach et al., 2018), and under a high-O_2_ atmosphere (Roach, 2020).

Gas chromatography-mass spectrometry (GC-MS) is also used for determining RCS and oxylipin carbonyls (Michalski et al., 2008). The exposure of *A. thaliana* plants to high light (1,500 μmol photons m^–2^ s^–1^) at low temperature (7°C) for 24 h increased the levels of 4-hydroxy-2-hexenal (HHE), HNE, and hexenal (Rac et al., 2021). The C3–C17 volatile compounds emitted during wounding were determined quantitatively in the leaves of five tropical crops (Portillo-Estrada et al., 2021). The medicinal plant *Rhodiola semenowii* when exposed to chilling stress, benzeneacetaldehyde and ketones such as 4-cyclopentene-1,3-dione and 1,2-cyclopentanedione were increased in the shoots; whereas plants under water-deficit stress showed decreased accumulations of ketones such as 2-propanone and 1-(acetyloxy)-2-propanone in the root (Terletskaya et al., 2021). Solid-phase micro-extraction fiber-trapped volatiles were measured by GC-time of flight (TOF)-MS and highly sensitive GC-quadrupole (Q)TOF-MS (Spyropoulou et al., 2017). GC-MS is also applicable for analyses of non-volatile oxylipin carbonyls after their derivatization (Kawai et al., 2007).

### Reactive Carbonyl Species Generation Mechanism

The endogenous level of an RCS is determined by the balance between its generation rate and the scavenging rate. We will explain the mechanism of RCS generation in plant cells and then describe the scavenging mechanisms for RCS.

RCS are formed through the oxidative degradation of LOOH. LOOH are generated by two distinct mechanisms: the enzymatic oxygenation of lipids, and the non-enzymatic oxidation of PUFAs by ROS. In an enzymatic process, lipoxygenase (LOX), a key enzyme of the oxidation of PUFAs such as α-linolenic acid, converts them to either 9- or 13-hydroperoxy-octadecatrienoic acids, or a mixture of both. Corresponding 9- and 13-hydroperoxide lyases act on 9- and 13-hydroperoxides, respectively, to form C6-C9 aldehydes and 9- and 13-oxoacid, including jasmonic acid and 12-oxo-phytodienoic acid (OPDA) (Matsui, 2006; Mosblech et al., 2009; Vincenti et al., 2019). OPDA has an α,β-unsaturated carbonyl structure and is therefore also an RCS. (*Z*)-3-Hexenal, the primary C6 product of the hydroperoxide lyase-catalyzed degradation of 13-hydroperoxide, is converted to (*E*)-2-hexenal, an RCS, by an isomerase (Kunishima et al., 2016; Spyropoulou et al., 2017). The enzymatic lipid oxidation and oxylipin metabolism in plants have been extensively studied as cellular responses relevant to infection and wounding.

The non-enzymatic lipid peroxidation process is explained in *in vitro* studies of oil deterioration under heat (Vliegenthart, 1979; Grosch, 1987). The highly oxidizing ROS, such as hydroxyl radical (HO^•^), starts the oxidation of a lipid molecule by abstracting a hydrogen atom from the pentadiene structure in a PUFA moiety, such as linoleic acid (18:2) and linolenic acid (18:3).

ROS are constitutively generated via the reduction of molecular oxygen (O_2_) in almost all of the subcellular compartments, among which chloroplasts and peroxisomes generate large fluxes of ROS formation in photosynthetic tissues under light, whereas the mitochondria contribute mainly to the flux in non-photosynthetic tissues (Foyer and Noctor, 2003; Mittler et al., 2004). In the chloroplast, the energy transfer from the triplet state of the primary electron donor (^3^P680*) in photosystem II (PSII) to ground state O_2_ generates ^1^O_2_, and the electron transfer from photosystem I (PSI)-ferredoxin to O_2_ produces O_2_^•–^ (Asada, 2006). Under high light conditions, ^1^O_2_ occupies a major portion of the ROS generated in leaves (Triantaphylidès et al., 2008; Waszczak et al., 2018). O_2_^•–^ is also produced in the mitochondrial electron transport chain when electrons from complexes I and II are overloaded (Huang et al., 2016). In the presence of superoxide dismutase (SOD), O_2_^•–^ is disproportionated to form H_2_O_2_ and O_2_. In the peroxisome, H_2_O_2_ generated by the glycolate oxidase activity during photorespiration is also a major source of ROS in C3 plants (Foyer and Noctor, 2003). HO^•^ is formed via the Fenton reaction in any part of the cell when H_2_O_2_ is reduced by metal catalysts such as Fe^2+^ ion (Mittler, 2017).

The NADPH oxidase complex (also known as the respiratory burst oxidase homolog; RBOH) on the plasma membrane is another important source of ROS. The *A. thaliana* genome contains 10 genes for the RBOH isoforms, which are expressed in a tissue-specific manner (Chapman et al., 2019). RBOHs are latent under normal conditions and activated when the cell receives stimuli, e.g., a pathogen attack or developmental signal (Mittler et al., 2011; Waszczak et al., 2018). The RBOH complex then reduces O_2_ to O_2_^•–^ in the apoplast by consuming NADPH in the cytosol. The O_2_^•–^ is then converted to H_2_O_2_ and O_2_ by the SOD in the apoplast.

The ROS thus generated in the close vicinity of membranes will oxidize the PUFA part of lipids in the membranes. Radicals, i.e., HO^•^ and O_2_H^•^ (the protonated form of superoxide radical) are neutral species and are capable of hydrogen abstraction from lipid molecules. The generated lipid radical reacts with O_2_ and forms a lipid peroxyl radical, which then oxidizes neighboring lipid molecules to cause radical chain oxidation to lipid peroxidation (Grosch, 1987). In addition to the radical chain reaction, the addition of ^1^O_2_ to a PUFA also generates a LOOH (Triantaphylidès et al., 2008). The subsequent fragmentation of LOOHs by the enzymatic and non-enzymatic processes forms dozens of oxylipin carbonyls, including RCS of various carbon-chain lengths and different extents of oxygenation (Farmer and Mueller, 2013; Yalcinkaya et al., 2019a).

### Reactive Carbonyl Species Scavenging Compounds and Enzymes

Plant cells are enriched with non-enzymatic and enzymatic scavenging systems to control the endogenous levels of RCS. As low-molecular-weight scavengers, the thiol-compounds cysteine and the reduced form of glutathione (GSH) can efficiently trap RCS by forming an RCS-conjugate (Esterbauer et al., 1975). With aluminum (Al) stress treatment, transgenic *A. thaliana* plants with higher levels of GSH exhibited less increases in RCS and other oxylipin carbonyls in roots and showed higher tolerance to Al toxicity compared to the wildtype plants (Yin et al., 2017). GSH may be critical for the scavenging of certain types of RCS because plants have glutathione transferase (GST) isozymes that show narrow specificities to selected types of RCS (Mano et al., 2019b, described below). Polyphenols (which are rich in plants) are also candidate RCS scavengers. Certain types of polyphenols such as phloretin in tea leaf and apple fruit (Zhu et al., 2009), pelargonidin in brown rice (Colzani et al., 2018), and catechins in tea leaf (Sugimoto et al., 2021) can trap acrolein and HNE *in vitro*, but their *in planta* function to scavenge RCS has not been verified.

Enzymatic reactions to scavenge RCS and non-RCS carbonyls in plants are classified into five types (Figure 1D) as follows: (*i*) 2-Alkenal reductase (AER) (Mano et al., 2002, 2005), and alkenal/one oxidoreductase (AOR) (Yamauchi et al., 2011) specifically reduce the α,β-unsaturated bond of an RCS molecule to form the corresponding saturated species. This type of enzyme does not react with the carbonyl group. AER from *A. thaliana* shows a higher affinity to longer-chain RCS than smaller RCS such as acrolein. (*ii*) Aldo-keto reductases (AKR) catalyze the reduction of a carbonyl to alcohol (Oberschall et al., 2000). AKR isozymes have a relatively broad substrate specificity for various carbonyls (Yamauchi et al., 2011). (*iii*) Aldehyde dehydrogenase (ALDH) catalyzes the oxidation of a carbonyl to a carboxylic acid (Kotchoni et al., 2006). (*iv*) GST catalyzes the formation of the covalent bond between an electrophilic compound (such as an RCS) and GSH to detoxify the former (Gronwald and Plaisance, 1998). The specificity to the electrophile substrate differs by isozymes. In the *A. thaliana* genome, there are about 50 isogenes of GST. Ten of the 28 total Tau-class GST isozymes can recognize acrolein or HNE or both as the substrate (Mano et al., 2017, 2019b), suggesting that GST is also an important enzyme for RCS detoxification. (*v*) Aldehyde oxidase (AO) catalyzes the oxidation of a carbonyl to carboxylate using O_2_ as the electron acceptor (Srivastava et al., 2017). *A. thaliana* has four AO isozymes. An extensive comparison of the substrate specificity of these RCS-scavenging enzymes is available in a recent article (Mano et al., 2019b).

## Response of Plants to Exogenously Added Reactive Carbonyl Species

### Reactive Carbonyl Species Can Modify Proteins

As shown in Figure 1, RCS can form Michael adducts or Schiff’s base with proteins, resulting in the modification of the Cys, Lys, His, and amino groups. Saturated carbonyls are capable of Schiff’s base formation but do not form Michael adducts. In contrast, methylglyoxal, and glyoxal, which are dialdehydes generated mainly through sugar metabolism, appear to modify Arg residues preferentially on the target proteins (Armed et al., 2005; Gao and Wang, 2006). The protein modification by RCS is thus distinct from that by saturated carbonyls and dialdehydes. On the other hand, ROS can react with amino acid residues which are not targeted by carbonyls, e.g., Trp, Tyr, and Met. HO^•^ can oxidize any type of organic compounds indiscriminately (Møller et al., 2007; Parvez et al., 2018). In addition, metal centers such as 4Fe-4S center and heme iron in proteins are sensitive to O_2_^•–^ and H_2_O_2_, respectively (Halliwell and Gutteridge, 2015).

Regarding the known H_2_O_2_ sensor proteins such as OxyR in bacteria and Yap1 in yeast, a specific Cys residue(s) is oxidized by H_2_O_2_ to the sulfenic acid (Cys-SOH), causing protein structural changes that result in the alteration of their physiological functions (Reczek and Chandel, 2015). Redox-reversible Cys residues on a protein can thus be affected by both ROS and RCS, but the chemical structures of the reaction products are different (Figure 1A). The modification of Cys residues in a protein may result in either its inactivation or activation, depending on the protein and the modifying compound (Wible and Sutter, 2017).

### Toxicity of Reactive Carbonyl Species

Because of their reactivity, carbonyl compounds are generally toxic to living cells. Reynolds (1977) compared the toxicity of various carbonyl compounds to lettuce seeds and showed that RCS are more toxic than saturated carbonyls. For example, the IC50 concentration to inhibit seed germination was 0.043 mM for acrolein and 13.7 mM for propionaldehyde. The exposure of *A. thaliana* plants to volatile RCS such as acrolein, methylvinyl ketone (MVK), and (*E,Z*)-2,6-nonadienal (Alméras et al., 2003) or (*E*)-2-hexenal (Matsui et al., 2012) caused a decrease in PSII activity. Acrolein caused the inactivation of PSII under light in the cyanobacterium *Synechocystis* sp. (Shimakawa et al., 2013). The infiltration of HNE into tobacco leaves caused necrosis (Mano et al., 2005).

The toxicity of RCS can be attributed to the inactivation of certain target enzymes. Pea mitochondria, alternative oxidase (Winger et al., 2005), and lipoate enzymes such as the H-subunit of glycine decarboxylase complex (Taylor et al., 2002) are highly sensitive to exogenously added HNE. In spinach chloroplasts, the addition of RCS resulted in the inactivation of photosynthesis. A comparison of various RCS and oxylipin carbonyls of C3–C9 revealed that an RCS is more toxic than the corresponding saturated carbonyl (as shown for seed germination). The most toxic RCS was acrolein, followed by HNE (Mano et al., 2009).

### Alteration of Gene Expression by Exogenous Reactive Carbonyl Species

At lower doses, exogenously added RCS can alter plant gene expression. *A. thaliana* plants upon exposure to (*E*)-2-hexenal, activated a wide range of genes to protect against pathogen attack (Bate and Rothstein, 1998; Kishimoto et al., 2005). An exogenous application of low levels of MDA (4 μmol L^–*l*^ air volume) to *A. thaliana* strongly upregulated many defense genes against abiotic/environmental stress (e.g., *ROF1*, *XERO2*, and *DREB2A*) (Weber et al., 2004). Acrolein and MVK upregulated the pathogenesis-related gene *HEL* (*PR4*) in *A. thaliana* plants (Alméras et al., 2003). The addition of OPDA, a C12 RCS, to *A. thaliana* liquid culture induced a set of defense genes that are distinct from those activated by jasmonate and MeJA, which are non-RCS downstream products of OPDA (Taki et al., 2005). The fumigation of *A. thaliana* plants with an RCS of carbon-chain length 4–8 expressed heat stress-related genes (*HSFA2*, *MBF1c*) and drought stress-related genes (*DREB2A*, *ZAT*s) and thus enhanced abiotic stress tolerance (Yamauchi et al., 2015). Salt-sensitive glycophyte *A. thaliana* and its close relative *Eutrema parvulum*, a salt-tolerant halophyte, responded differently after exposure to various concentrations of RCS. Applications of acrolein, HHE, and HNE increased the activity of various ROS scavenging enzymes such as catalase and ascorbate peroxidase in *A. thaliana* but not in *E. parvulum.* The gene expressions of the membrane-bound *SOS1* and tonoplast-localized *NHX1* and *NHX5* were upregulated by RCS, depending on the RCS type and concentrations (Yalcinkaya et al., 2019b).

In mammals, transcription factor Nrf2 is a master regulator of cellular responses against environmental stressors, which induces the expression of detoxification genes. Under normal conditions, the Nrf2 level in the cell is kept low by the action of Keap1 (Kelch-like ECH-associated protein 1), an adaptor subunit of Cullin 3-based E3 ubiquitin ligase. Keap1 catalyzes the ubiquitination of Nrf2 and facilitates its degradation. Under oxidative stress, HNE modifies Keap1 on its Cys residues 151, 273, and 288 and inhibits its ubiquitin ligase activity. HNE thus raises the Nrf2 level and thereby activates the target genes (Suzuki and Yamamoto, 2017). A similar RCS-mediated gene regulation mechanism may well be present in plants. The exposure of *C. reinhardtii* cells to a low concentration of acrolein (600 ppm) increased the glutathione content and the expression of the GST isogene *GSTS1*. These responses protected the algal cells from ^1^O_2_ generated under high light (Roach et al., 2017). The ^1^O_2_-tolerance was induced by acrolein at as low as 125 ppm, while acrolein at higher than 900 ppm caused damage to cells, representing a typical continuum of the effect of RCS from eustress to distress (Roach et al., 2018). The 8 base-pair palindromic sequence CAACGTTG (electrophile-responsive element; ERE) in the promoter region is required for the (*E*)-2-hexenal-responsive expression of *GSTS1* (Fischer et al., 2012) and other defense genes including *IFR1* (Venkanna et al., 2017) in *C. reinhardtii*. SINGLET OXYGEN RESISTANT 1 (SOR1), a bZIP-type putative transcription factor, is a candidate to be bound to ERE as a repressor because the *SOR1*-deficient mutant *sor1* shows overexpression of the electrophile-responsive genes (Fischer et al., 2012). It has yet to be clarified whether the SOR1 protein is the (*E*)-2-hexenal sensor or additional factors mediate the RCS signal to SOR1.

## Physiological Roles of Endogenously Generated Reactive Carbonyl Species That Are Verified by the Measurement of Reactive Carbonyl Species Contents

### Reactive Carbonyl Species Are Causative Agents of Oxidative Injury in Plants

In the early 2000s, pioneering work was published regarding the damage-causing effect of endogenously produced carbonyls, in which the overexpression of carbonyl-scavenging enzymes such as ALDH and AKR conferred tolerance against environmental stressors in transgenic plants (Oberschall et al., 2000; Sunkar et al., 2003; Hegedüs et al., 2004; see Table 1). The toxicity of the endogenous carbonyls was further supported by the observations of the transgenic plants with suppressed ALDH levels. When the expressions of *ALDH7* and *ALDH3* genes were suppressed in *A. thaliana*, the plants showed greater sensitivity to NaCl (Kotchoni et al., 2006). Similarly, the suppression of *ALDH7* expression in rice made the plants hypersensitive to NaCl and chilling (Shin et al., 2009). The silencing of *NbALDH2C4* in *Nicotiana benthamiana* enhanced the sensitivity of the plants to low temperature with higher accumulations of ROS and MDA, and in potatoes, the overexpression of *ALDH2B7a* lowered the level of aldehydes and enhanced the cold stress tolerance (Guo et al., 2020). In these studies, the TBARS level and the extent of tissue injury were shown to be correlated in both wildtype and transgenic lines. This evidence strongly supported the participation of the endogenous oxylipin carbonyls in the injury, but it was unclear which carbonyl species were increased and damaged the cells.

HPLC-based comprehensive analyses of carbonyls identified the carbonyl species involved in tissue injury, as follows (Mano et al., 2010; Yin et al., 2010; Kai et al., 2012; Yamauchi et al., 2012; see Table 1). The application of photoinhibitory intensity light to wildtype tobacco leaves caused significant accumulations of RCS such as acrolein, (*E*)-2-pentenal, and (*E*)-2-hexenal prior to the appearance of photoinhibition symptoms; whereas transgenic tobacco plants that overproduced AER accumulated lower levels of these RCS, and eventually their leaves suffered less damage (Mano et al., 2005, 2010). Conversely, the knock-out of chloroplastic AOR in *A. thaliana* resulted in hypersensitivity to methylviologen, in association with a higher accumulation of acrolein (Yamauchi et al., 2012).

The treatment of tobacco roots with aluminum chloride (AlCl_3_) increased the ROS level in the elongation zone and eventually caused cell death (Yamamoto et al., 2002). The AlCl_3_ treatment also increased the RCS acrolein, HNE, and HHE by 1.2 nmol (g tissue)^–1^ and also less-reactive non-RCS carbonyls; for example, formaldehyde was increased by 40 nmol (g tissue)^–1^. In contrast, in AER-overexpression (OE) plants, the AlCl_3_-induced increases of RCS and non-RCS carbonyls were significantly suppressed and the transgenic plants suffered less injury by the Al ion (Yin et al., 2010). Importantly, the absorption of Al ion and the enhancement of the ROS level at the target site (the elongation zone) in the AlCl_3_-treated roots were not affected by the overexpression of AER. These results support the possibility that the protection of cells in AER-OE plants is attributable solely to RCS scavenging. In other words, the RCS produced downstream of ROS caused tissue injury.

Heat-stress treatment of wildtype cyclamen plants (*Cyclamen persicum* Mill.) resulted in accumulations of (*E*)-2-hexenal, acrolein, and MVK, and the leaves were consequently damaged. An RNAi-suppression of *CpFAD7* (chloroplastic fatty acid desaturase 7) in cyclamen plants resulted in lower trienoic fatty acids contents and thus less accumulation of these three RCS. These RNAi lines did not show any visible damage symptoms under heat stress (Kai et al., 2012). The formation of these RCS was therefore related to the heat-induced damage in the leaves.

### Signaling Functions of the Endogenously Generated Reactive Carbonyl Species

The signaling functions of endogenously generated RCS have also been verified by the measurement of RCS contents and by the modulation of RCS levels with a scavenging enzyme or a chemical scavenger, as follows (Biswas and Mano, 2015; Islam et al., 2016, 2020; Srivastava et al., 2017; Biswas et al., 2019; see Table 1). Programmed cell death (PCD) is a genetically regulated process that directs a cell to eliminate itself in an organized way, as a strategy used by plants to survive stressful conditions. The cells damaged by biotic or abiotic stressors accumulate ROS, which accelerates the PCD process (Petrov et al., 2015). For our studies of ROS signaling mechanisms, we used H_2_O_2_-induced PCD in tobacco BY-2 cells, an experimental model system, and we tested the hypothesis that RCS are involved in the ROS signaling for initiating PCD (Biswas and Mano, 2015). The addition of H_2_O_2_ to BY-2 cells caused increases in RCS such as acrolein, HNE, and HHE and non-RCS carbonyls such as acetaldehyde, propionaldehyde, and *n*-hexanal within 2 h, while typical PCD symptoms (TUNEL-positive nuclei, DNA laddering, and cytoplasm shrinkage) appeared 5 h after H_2_O_2_ treatment. The addition of the chemical carbonyl scavengers carnosine and hydralazine suppressed increases in both RCS and PCD symptoms in H_2_O_2_-treated cells, without affecting the levels of ROS and LOOH in the cells. Therefore, these chemicals suppressed the H_2_O_2_-induced PCD by scavenging RCS. Our investigation also demonstrated that the overexpression of AER in tobacco prevented root epidermis PCD under salt stress (Biswas and Mano, 2015). Specifically, the RCS generated in the ROS-stimulated cells acted as the signal to turn on the death program of plant cells.

Senescence is the aging process of various parts of the plant body, and internal and external stimuli initiate the cell death program characterized by the expression of senescence-related genes. RCS were also shown to be involved in the senescence of Arabidopsis siliques, as follows. The Arabidopsis aldehyde oxidase 4 (AAO4) can detoxify RCS and non-RCS carbonyls. The siliques of knockout mutant *aao4* accumulated higher levels of acrolein and MDA in darkness and a higher level of MDA after UV-C exposure compared to the wildtype and showed accelerated senescence (Srivastava et al., 2017). The involvement of RCS in the senescence was further supported by the observation that exogenously added acrolein and HNE enhanced silique senescence in *aao4*, but not in the wildtype.

Functions of RCS in hormone signaling are also being revealed. ROS is involved in auxin-triggered lateral root (LR) formation (Orman-Ligeza et al., 2016), but the ROS signaling mechanism had been poorly understood. We observed that the addition of auxin to *A. thaliana* roots caused increases in the levels of various oxylipin carbonyls (such as HNE, crotonaldehyde, formaldehyde, and butyraldehyde) prior to the apparent LR emergence. Importantly, the simultaneous addition of the carbonyl scavenger carnosine and auxin to roots suppressed both the increase in the carbonyl levels and LR formation. When an RCS such as acrolein or crotonaldehyde was added to roots, the degradation of the auxin signaling repressor Aux/IAA protein was facilitated, and the auxin-responsive genes for LR formation were activated at the LR-forming sites (Biswas et al., 2019). These results demonstrate that RCS reinforce the auxin signaling for LR formation, by facilitating the degradation of Aux/IAA.

In the stomata closure induced by abscisic acid (ABA) and methyl jasmonate (MeJA), the production of ROS in guard cells is involved as a signal (Pei et al., 2000; Suhita et al., 2004). The addition of H_2_O_2_ and ABA (Islam et al., 2016, 2019) and MeJA (Islam et al., 2020) to leaf epidermis increased the levels of acrolein, HNE, and HHE; the addition of HNE to leaf epidermis caused stomata closure (Islam et al., 2016) preceded by the typical [Ca^2+^] elevation in the guard cells (Islam et al., 2019). The AER-OE tobacco leaves accumulated fewer RCS in response to MeJA, ABA, and H_2_O_2_ and exhibited smaller stomatal closure responses. These findings indicate that RCS generated in the guard cells, downstream of H_2_O_2_, acted as a signal for stomatal closure in the ABA and MeJA signals.

The above-described results suggest that RCS are critical signaling molecules involved in plant growth and development and cell death. However, to elucidate the functional mechanisms of RCS, it is necessary to determine which carbonyl is the most important player for each RCS-induced response. Considering the evidence obtained thus far, we can see that different physiological responses have different requirements for carbonyls. For example, the heat-shock response genes were induced by fumigation with RCS of C4–C8 chain length but the C3 RCS acrolein did not exert this effect (Yamauchi et al., 2015). For the promotion of LR formation, strong effects were observed for the RCS acrolein, HNE, and crotonaldehyde, in that order. Interestingly, butyraldehyde and propionaldehyde, which are non-RCS carbonyls, also induced LR formation at higher doses than RCS. In contrast, *n*-hexanal, another non-RCS carbonyl, inhibited LR formation (Biswas et al., 2019).

Not only the strength but also the intracellular level of a carbonyl will determine its contribution to plant responses. In Al-stressed roots, many types of RCS and non-RCS carbonyls were increased. The formaldehyde content in the stressed cells was 100-fold higher than the acrolein content (Yin et al., 2010). Considering that formaldehyde is 400-fold less toxic than acrolein (Reynolds, 1977), we can estimate the former’s contribution is 1/4 of the latter. Similarly, in H_2_O_2_-stimulated BY-2 cells, multiple types of RCS and non-RCS carbonyls were increased. In the stressed cells, acrolein was increased to 2.5 nmol/g cells, whereas n-hexanal was increased to 18 nmol/g cells. Because the PCD-inducing strength of acrolein is 15-fold higher than that of n-hexanal (Biswas and Mano, 2015), the contribution of n-hexanal is estimated as roughly half of that of acrolein. The significance of non-RCS carbonyls is therefore not negligible, at least in these responses.

## Target Proteins of Reactive Carbonyl Species

Once the involvement of RCS in a physiological phenomenon (e.g., metabolic pathways such as respiration, or signaling pathways such as ABA-induced stomata closure) is identified, the next step for the elucidation of the mechanism is to determine the target protein that is primarily modified by the RCS. There are two approaches for the identification of target proteins: the functional approach and the comprehensive approach.

### Functional Approach

In the functional approach, the number of candidate RCS target proteins in the RCS-sensitive biochemical pathway of interest is narrowed down by examining the RCS effect on partial reactions of distinct enzymes. Millar and Leaver (2000) observed that the addition of HNE to potato mitochondria inactivated respiration, and they identified the most sensitive target as glycine decarboxylase complex. Taylor et al. (2002) revealed that HNE was covalently bound to the lipoic acid in the active site of the H protein of the complex and blocked the catalytic activity. In the mitochondrial inner membrane, uncoupling protein 1 was observed to be sensitive to HNE (Winger et al., 2005). Regarding photosynthesis, the addition of acrolein to spinach chloroplasts inactivated the CO_2_-fixation activity, whereas the thylakoid electron transport chain was not affected (Mano et al., 2009). Specifically, the primary target site was in the Calvin cycle, and it was finally revealed that the thiol-regulated enzymes phosphoribulokinase, fructose-bisphoshatase, and glyceraldehyde-3-phosphate dehydrogenase were the sensitive targets. On the other hand, acrolein added to Synechococcus cells under light caused the inactivation of the PSII reaction. A proposed mechanism is that the photoproduced HO^•^ reacted with acrolein and was bound to the PSII complex (Shimakawa et al., 2013).

Wounding treatment in jasmonate biosynthesis-deficient *A. thaliana* mutants showed that OPDA, a precursor RCS to jasmonate, functions as a signaling molecule for diverse physiological processes in plants (Taki et al., 2005). OPDA also repressed cell-cycle regulation and cell growth in Arabidopsis cell cultures (Eckardt, 2008) and suppressed the root growth induced by TGA transcription factors (Stotz et al., 2013). It has been suggested that cellular GSH and redox potentials play a potential role in OPDA signaling, and cyclophilin 20-3 was shown to bind OPDA to maintain the cellular redox homeostasis in stress responses via the formation of cysteine synthase complex (Park et al., 2013).

RCS are involved in the ABA- and MeJA-dependent signaling for stomata closure (Islam et al., 2016, 2020). The action site of RCS in the ABA signaling pathway should reside upstream of the activation of the putative Ca^2+^ channel because exogenous addition of acrolein stimulated the Ca^2+^ influx into the guard cell (Islam et al., 2019). Interestingly, the Arabidopsis mutant *tgg1tgg2*, deficit of two myrosinases TGG1 and TGG2, was insensitive to acrolein with regard to the stimulation of Ca^2 +^ influx, indicating that these myrosinases are required for the acrolein signaling (Rahman et al., 2020). It has yet to be clarified whether the myrosinases are the RCS targets.

In tobacco BY-2 cells, the addition of the PCD-initiating level of H_2_O_2_ stimulated the activities of caspase-1-like protease (C1LP) and caspase-3-like protease (C3LP), which are cysteine proteases that are known to trigger PCD in plants (Ye et al., 2013; Hatsugai et al., 2015). A lethal dose of acrolein increased the activity of C3LP (Biswas and Mano, 2016). Acrolein and HNE added to cell-free extracts stimulated the C3LP and C1LP activities, but H_2_O_2_ could not (Biswas and Mano, 2016). These proteases are thus direct targets of RCS. C3LP activity is displayed by two different proteins: the β1 subunit of 20S proteasome (Hatsugai et al., 2015) and cathepsin B (Ge et al., 2016). The RCS-responsive C3LP activity has been attributed to the former protein on the basis of an inhibitor study (Biswas et al., 2020).

In the auxin signaling for LR formation, RCS promote the degradation of the Aux/IAA repressor protein, which is a key step in auxin signaling (Biswas et al., 2019). In the *slr1* mutant of *A. thaliana* (which does not form LRs because an amino acid change in IAA14 protein prevents its degradation by the proteasome; Fukaki et al., 2002), RCS was ineffective to promote LR formation. These results indicate that the primary RCS action site resides in-between the auxin recognition by the receptor TIR1 and the degradation of Aux/IAA (Biswas et al., 2019). One possible target is TIR1. Cys140 in the TIR protein can be modified by nitric oxide (NO), and this modification increases the affinity of the TIR1-Aux/IAA complex to auxin. This explains NO’s ability to promote LR formation (Terrile et al., 2012). Because both NO and RCS are electrophiles, RCS may be bound to the same Cys residue and facilitate the auxin signaling by the same mechanism. Alternatively, IAA14 protein may be the RCS sensor.

### Comprehensive Approach

In the comprehensive approach, RCS-modified proteins are identified by proteomic analysis. The plants or cells of interest are treated with oxidative agents, and the RCS-modified proteins in cell extracts are marked or collected by an anti-RCS antibody for the subsequent proteomic analysis. Winger et al. (2007) detected HNE-modified proteins in *A. thaliana* mitochondria by western blotting with an anti-HNE antibody after two-dimensional electrophoresis. The immunoreactive proteins increased by the treatment of cells with H_2_O_2_, antimycin A, and menadione were extracted from the gels and sequenced. The list of the HNE-modified proteins in oxidative stressed cells largely overlapped with the proteins in the cells treated directly with HNE, indicating that RCS preferentially modifies certain proteins.

Mano et al. (2014) collected HNE-modified proteins by antibody affinity trapping from the leaves of *A. thaliana* and compared the amount of each of the proteins between the NaCl-stressed samples and the control samples by a differential quantitative proteomics technique. Seventeen proteins were observed to be more frequently (more than twofold) modified with the endogenously produced HNE under salt stress. Based on the identities of these target proteins, it was observed that under NaCl stress, HNE affected proteins in the cytosol, peroxisome, chloroplast, mitochondrion, and (interestingly) apoplast. This intracellular distribution of the HNE target proteins suggests that under stress conditions, membranes of various organelles and the plasma membrane are oxidized and produce RCS.

An advanced comprehensive analysis has been applied to investigate the various types of oxidative modifications of proteins in legume nodules (Matamoros et al., 2018). In this method, a carbonyl moiety on a protein, which is formed due to oxidation or RCS addition (Møller et al., 2007), is labeled with a biotin probe. The labeled protein fragments are collected by avidin affinity chromatography and then analyzed with LC-ESI-Orbitrap-MS, which allows identification of the modified peptide, the modified amino acid residue, and the type of carbonylation. In the root nodules, 12 types of carbonylations affecting six amino acid residues were identified in 238 proteins. Interestingly, Lys was the most commonly carbonylated amino acid and accounted for 58% of all modifications, followed by His, Cys, Arg, Thr, and Pro (Matamoros et al., 2018). It is also possible to investigate the difference of the extent of modification extent on the same target protein in samples treated differently. This method has a great potential to investigate the RCS target proteins that are involved in plant responses to oxidative stimuli.

The comprehensive approach is useful for creating a list of potential targets of RCS, but the significance of the RCS modification or each target protein should be investigated in separate experiments. An examination of the effects of RCS on the biochemical activity of the purified target protein should be conducted, and in clarifying the physiological significance of the RCS modification of a target protein, a genetic analysis using a mutant is preferable.

## Discussion

As described thus far, the significance of RCS as signal mediators downstream of ROS is evident. There is an open question about the ROS signaling mechanism: how can ROS, the only four species, transmit signals to specific targets in different physiological situations? (Møller and Sweetlove, 2010). The variety of RCS species, which is greater than that of ROS, may provide a clue to this specificity problem if the relationships between the ROS types and the downstream RCS types are revealed. Because RCS have comparatively longer lifetimes than ROS (Parvez et al., 2018), they are suitable for conveying signals within a cell and between cells. Considering the critical significance of ROS in a wide range of physiological phenomena in plants from cell division to pollination, seed germination to fruit ripening (Mittler, 2017; Mhamdi and Van Breusegem, 2018; Waszczak et al., 2018), and stress responses (Gill and Tuteja, 2010; Schippers et al., 2016), further explorations of the physiology of RCS will surely uncover many interesting facts.

For investigations of the involvement of RCS in a physiological phenomenon, a recommended starter experiment is to examine the effect of carbonyl scavengers on the phenomenon, as was done for PCD in BY-2 cells (Biswas and Mano, 2015). If RCS are involved, the increase in the endogenous RCS levels should be associated with the examined phenomenon, and the addition of carbonyl scavengers should suppress the RCS levels and the response of the cell. Because carnosine reaction has the bias for distinct RCS, it is necessary to clarify which carbonyl species are decreased. One important precaution is that most of the compounds known as carbonyl scavengers, e.g., the dipeptide carnosine, may have antioxidant effects (Aldini et al., 2007; Zhu et al., 2011). Thus, if the scavenger suppresses the tested phenomenon, it is necessary to confirm that the ROS levels are not affected. The LOOH level test with the fluorescent dye Spy-LHP (Khorobrykh et al., 2011) will provide another negative control for determining whether or not a carbonyl scavenger affects the formation of LOOHs.

For further examinations of the RCS function in the above-verified phenomenon, analyses of the carbonyls and the identification of the target proteins is necessary. The HPLC (or LC-MS) analysis of carbonyls allows the simultaneous determination of multiple types of carbonyls in one sample, but it requires the extraction of carbonyls from samples of at least tens of mg fresh weight of plant tissue. This requirement practically limits the carbonyl analysis in a specialized type of cells such as guard cells or the LR primodia cells. The visualization and detection of carbonyls in the tissues and distinct cells by chemical probe-based observations or a mass spectrometry imaging system would be a critical technique in the understanding of RCS physiology in plants. The identification of target proteins also requires certain amounts of samples. The most advanced single-cell proteomics (Kelly, 2020) in combination with proper methods for the in situ labeling of the modified proteins as described above (Matamoros et al., 2018) may provide a breakthrough.

Last but not least, there is an almost untouched but very critical issue regarding RCS: the relationship of RCS signaling with other reactive species, i.e., ROS, reactive nitrogen species (RNS), and reactive sulfur species (RSS). Nitric oxide has an electrophilic nature and can form a covalent bond with the thiol group. Specifically, RCS and NO may compete for Cys on the target proteins. RNS can react with lipids to form nitro-fatty acids (Sánchez-Calvo et al., 2013), but their metabolism and physiological significance in plants have scarcely been investigated. RSS include hydrogen sulfide and persulfides (R-SSH), which are endogenously produced and act as signaling molecules (Aroca et al., 2018). Regarding the chemical properties of RSS, they are nucleophiles as potent as thiol compounds (Benchoam et al., 2020), and accordingly, they may modulate RCS signals. Conversely, RCS may modulate RSS signaling. These reactive species, i.e., ROS, RNS, RCS, and RSS (RONCSS; De Tullio and Asard, 2012) are critical factors for determining cell fates, because not only are they bioactive agents but also do they react with antioxidants to alter the redox status of the cell in a rapid and microlocal manner, which may eventually affect the global redox responses of cells in the tissue. It will be a challenging task to delineate the cellular logic of reactive molecules, in which all RONCSS in the same samples are comprehensively analyzed.

## Author Contributions

JM conceived and supervised the review topics. MB wrote the first draft. Both authors contributed to the article and approved the submitted version.

## Conflict of Interest

The authors declare that the research was conducted in the absence of any commercial or financial relationships that could be construed as a potential conflict of interest.

## Publisher’s Note

All claims expressed in this article are solely those of the authors and do not necessarily represent those of their affiliated organizations, or those of the publisher, the editors and the reviewers. Any product that may be evaluated in this article, or claim that may be made by its manufacturer, is not guaranteed or endorsed by the publisher.

## Footnotes

1 The term “RCS” sometimes includes, in addition to the LOOH-derived α,β-unsaturated carbonyls, the sugar-derived dicarbonyls methylglyoxal and glyoxal (a broad definition of RCS). In this review article, however, we adopt a narrow definition of RCS; we exclude these dicarbonyls because we believe that they should be distinguished from oxylipin-derived carbonyls in terms of both physiology and chemistry. A review article is available on these sugar-derivided dicarbonyls in plants (Hoque et al., 2016).
